# Temporary Frontal Paralysis Secondary to Blunt Trauma Frontal Sinus Fracture

**DOI:** 10.1155/2017/4268259

**Published:** 2017-05-10

**Authors:** Mark Bastianelli, Stefan Hamilton, Matthew Hearn, Safeena Kherani, Kristian I. Macdonald

**Affiliations:** Department of Otolaryngology-Head and Neck Surgery, University of Ottawa, The Ottawa Hospital, Ottawa, ON, Canada

## Abstract

Frontal sinus fractures (FSF) are relatively uncommon and can be challenging for trauma surgeons to manage. Patients with FSF typically present with facial swelling, pain, and nasofrontal ecchymosis. Here we present a rare case of a patient with FSF and anterior table fracture where the main presenting symptom was bilateral frontal paralysis. We outline our management strategy and review the current literature in regard to management of FSF.

## 1. Introduction

Frontal sinus fractures (FSF) are relatively uncommon and can be associated with significant morbidity [1]. Etiologies of FSF include high-energy, blunt traumatic events such as motorcycle accidents, automobile accidents, and interpersonal violence and are often associated with multiple concomitant injuries [2]. Up to 66% of patients will have associated facial fractures [3].

Symptoms of patients presenting with frontal sinus fracture include frontal pain, edema, and ecchymosis [1, 2, 4]. More severe comminuted anterior table fractures can result in facial deformity and may necessitate surgical correction. Posterior table involvement is associated with more serious complications including intracranial hemorrhage, pneumocephalus, cerebrospinal fluid (CSF) rhinorrhea, blocked frontal outflow tract, and meningitis [4, 5].

We present a rare case of blunt forehead trauma and anterior table fracture in which frontal paralysis was the major presenting symptom. This is not a typically described sign of FSF, and our experience may help other clinicians who could be faced with this situation. In the following report we outline our case, describe our management, and review the literature for similar cases.

## 2. Case Presentation

A healthy 15-year-old male presented to the emergency department after sustaining a head injury during a soccer tournament. The patient described colliding with another player while heading a soccer ball. The patient denied any immediate loss of consciousness, distorted awareness, blurred vision, epistaxis, or otorrhea. His main concern was pain and swelling over the forehead as well as an appreciable defect/dip in the normal continuity of his frontal sinus bone.

**Figure 1:**
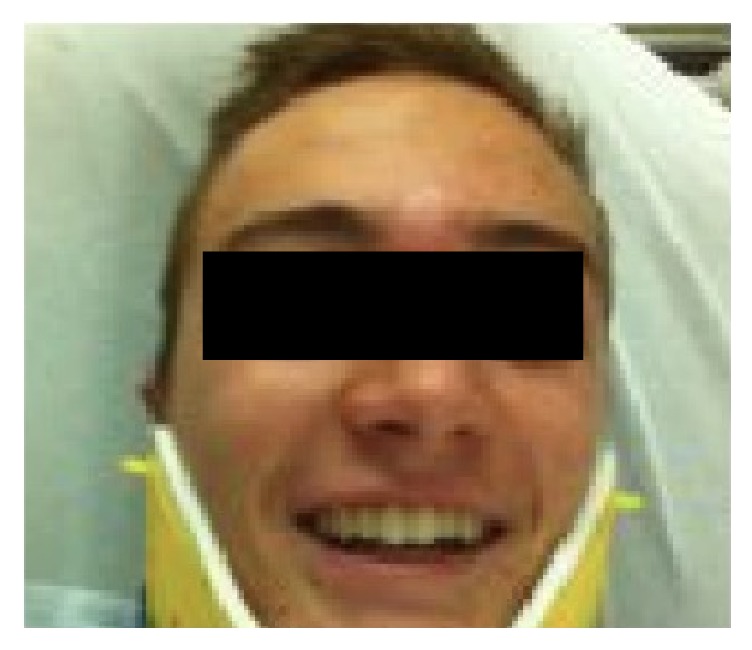
Early postinjury view showing acute glabellar ecchymosis and swelling.

On initial examination the patient appeared well and in no sign of distress. There was substantial edema and an appreciable convexity in the frontal sinus bone directly above the glabella (Figure 1). His facial expression appeared flat, and there was an obvious bilateral paralysis of the frontalis muscle (temporalis branch of CN VII). All other CN VII branches were intact. The nasal bones appeared and felt intact with no evidence of fracture or crepitus. On anterior rhinoscopy, there was a mild, nonobstructing, left sided septal deviation with no evidence of active bleeding or septal hematoma.

**Figure 2:**
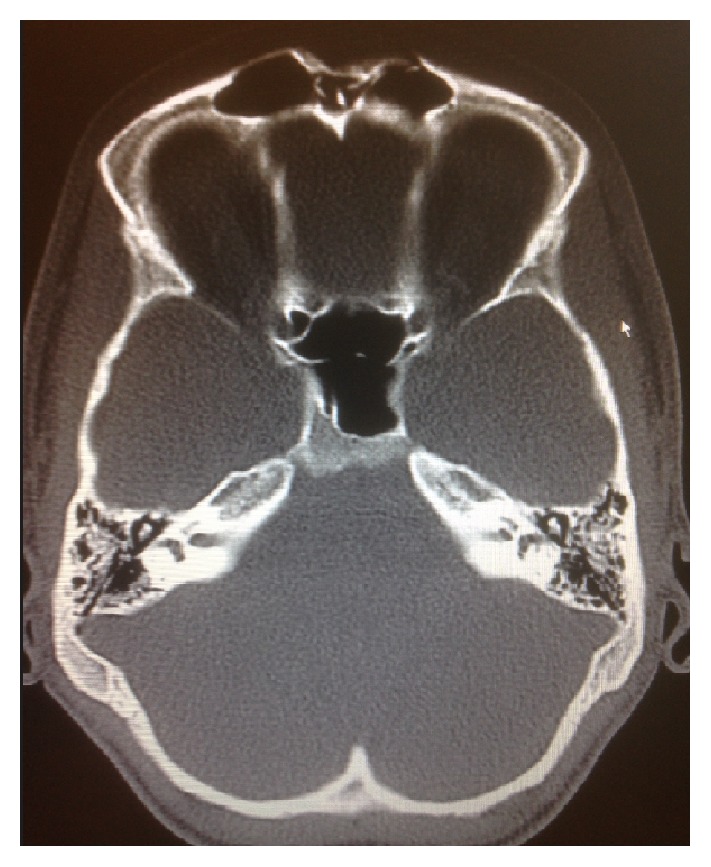
CT head demonstrating comminuted depressed fracture involving anterior wall of bilateral frontal sinuses and mildly displaced fracture of the nasal bones.

While in the emergency department the patient had a CT head, which showed a comminuted, depressed fracture involving anterior wall of bilateral frontal sinuses and mildly displaced fracture of the nasal bones (Figure 2).

**Figure 3:**
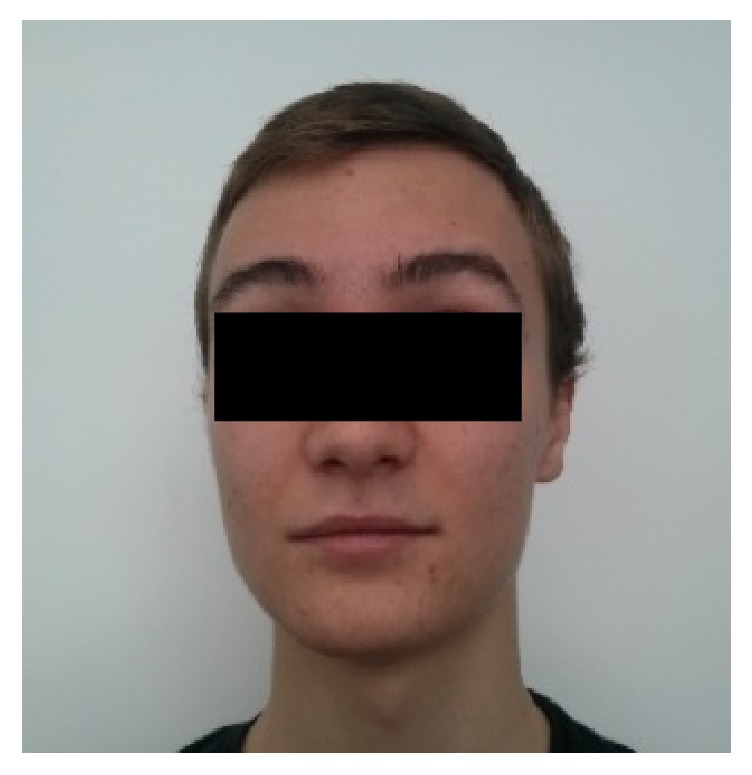
Resolved paralysis with normal brow elevation at week 8.

The patient was referred to an Otolaryngologist at a tertiary care centre. After consultation with the family and because the patient was otherwise stable, we agreed to manage the forehead paralysis with observation and close follow-up. Fortunately, after 8 weeks the patient's forehead paresis fully recovered with this approach (Figure 3).

## 3. Discussion

Frontal sinus fractures are not common in maxillofacial injuries; likely in part because it is so well protected by thick cortical bone, the frontal sinus is rarely fractured. In fact, the frontal bone is more resistant to fracture than any other facial bone, occurring in only 5–12% of all maxillofacial injuries [4]. Normally, a substantial amount of energy is required to cause a fracture, with the most common scenario being a motor vehicle accident (MVA). Demographically, the literature reveals a predisposition towards frontal sinus fractures among white males between the ages of 30 and 34 [2, 4].

Frontal sinus fractures are classified by their location within the sinus. A recent retrospective analysis notes that 62% of fractures involve both the anterior and posterior tables of the sinus, 31% involve the anterior table only, and 7% involve the posterior table. Concomitant nasofrontal duct injury is also important to document, occurring in 70% of all fractures [4]. Differentiating between anterior and posterior table fractures is vital for patient prognosis and management. For example, a mild anterior table fracture may only result in mild frontal pain and edema, whereas a fracture involving both the anterior and posterior tables may produce severe cosmetic deformity, intracranial hemorrhage, CSF fistula, and pneumocephalus. If the nasofrontal outflow tract is injured and becomes obstructed, the sinus can become a nidus for chronic infection and mucocele formation [5–7].

Diagnosis of frontal sinus fracture is best made with a careful history and physical exam and with computed tomography (CT) of the facial bones and paranasal sinuses. Coronal and sagittal reconstructions are necessary to characterize any fracture displacement or comminution [8].

The management strategy for frontal sinus fracture is variable and depends on which structures are involved [4, 6]. In the case of a pure, minimally displaced anterior table fracture, simple observation is recommended. If this is complicated by a minor contour deformity, injectable fillers such as calcium hydroxyapatite or poly-I-lactic acid can be used; larger deformities can be addressed using open reduction and internal fixation. If the nasofrontal duct becomes obstructed, obliteration of the sinus was an acceptable management option; however, with our current technical ability and instrumentation in sinus surgery, it may be preferable to attempt to open the tract endoscopically. In the case of a nondisplaced posterior table fracture, surveillance with interval CT scans is acceptable [9]. If the posterior table is displaced, cranialization with removal of the posterior table is classically indicated in order to avoid serious intracranial complications. Recent evidence, however, demonstrates that displaced posterior table fractures, even when there is an associated CSF leak, may be safely managed without surgical intervention [4]. It is suggested that a trial of observation is a safe initial approach. Endoscopic salvage procedures are highly efficacious if complications arise during this initial observation period [9]. Cranialization should instead be reserved for severely comminuted posterior table fractures, as this intervention carries a risk for long-term complications such as chronic headache, sinusitis, and altered sensation in the ophthalmic distribution.

Frontalis weakness in the setting of a blunt trauma frontal sinus fracture is a rare finding. In an 11-year retrospective review by Fox et al., one patient who underwent cranialization for a fracture involving displacement of both anterior and posterior tables complained of frontalis weakness on the affected side [4]. He also complained of chronic headaches and decreased sensation in the ophthalmic distribution of the affected side. His initial postoperative recovery had been uneventful. The authors do not speculate as to the etiology of the frontalis weakness; they only conclude that cranialization may not be appropriate for minimally displaced posterior table fractures based on noninferior outcomes in similar patients who were managed nonoperatively. A second retrospective review by Strong et al. analyzed 130 patients who were treated surgically for frontal sinus fracture over 15 years and found that 5 patients (4%) suffered from temporal nerve paresis postoperatively [1]. Unfortunately, no information was given on the severity of the fracture, the type of surgery, or the timing of the complication.

Fortunately, the literature on traumatic injury to the facial nerve is more abundant and offers some relevant guidelines. In a review by Lee et al., the authors advocate expectant management for blunt trauma to the extratemporal facial nerve, stating that surgery should only be considered if there is no recovery after 6 months [10]. Specifically, they state that blunt injury to the nerve medial to the lateral canthus often resolves spontaneously due to its rich cross-anastomotic connections in that region. Another study describes two separate cases of facial weakness secondary to blunt trauma to the extratemporal facial nerve [11]. Both patients were managed with a short course of high-dose corticosteroids and both recovered fully in 3 months or less. The authors concede that the role of steroids in this scenario is uncertain because of insufficient evidence. They instead conclude that recovery is most likely spontaneous and there is no clear indication for surgical management of the nerve.

Our patient presented with a comminuted fracture involving the anterior table of both frontal sinuses and associated bilateral frontalis paralysis. The patient was observed and reassessed in 6 weeks, at which time the weakness had completely resolved. Although it is unclear whether there was an injury to the facial nerve or impaired muscle movement secondary to edema, we feel that expectant management was appropriate in this case. There are no truly analogous cases reported in the literature but the guidelines regarding management of blunt trauma to the extratemporal facial nerve are consistent with our approach.

## 4. Conclusion

Frontalis weakness is a rare complication of frontal sinus fracture. As with any frontal sinus fracture, it is important to systematically assess any injuries to the anterior table, posterior table, and nasofrontal duct. In the stable patient with an isolated anterior table fracture with no other complications, simple observation is a safe initial strategy to manage frontalis weakness.
